# A Middle-aged Woman with a Persistent Gastrointestinal Bleed

**DOI:** 10.4103/1319-3767.80389

**Published:** 2011

**Authors:** Turki AlAmeel, David K. Driman, Richard P. Reynolds

**Affiliations:** Department of Medicine, London Health Science Center, The University of Western Ontario London, Ontario, Canada; 1 Department of Pathology, London Health Science Center, The University of Western Ontario London, Ontario, Canada

A 59-year-old female with a history of hypothyroidism and bronchial asthma presented to the emergency department with a 1 day history of hematemesis. She was well until 1 week prior to presentation when she developed profound fatigue and melena stool. There was no history of diarrhea or dyspepsia. Her medications included L-thyroxine, salbutamol inhaler PRN, and oral pantoprazole that was started 3 days prior to her hospital visit. She denied using aspirin, nonsteroidal anti-inflammatory drugs (NSAIDs), or excessive alcohol.

Physical examination revealed pallor and a mild orthostatic drop in blood pressure. Her hemoglobin was 55 g/L and other routine blood tests were normal. Esophagogastroduodenoscopy showed a clot at the lesser curvature and a clean-based ulcer at the pylorus. She was started on intravenous pantoprazole and given blood transfusion and supportive treatment. The patient continued to bleed and repeat esophagogastroduodenoscopy 2 days later showed healing of the pyloric ulcer and diffuse gastritis; multiple biopsies were taken from the body and antrum of the stomach. Biopsies were negative for *Helicobacter pylori* and showed the following [Figure 1].

**Figure 1:**
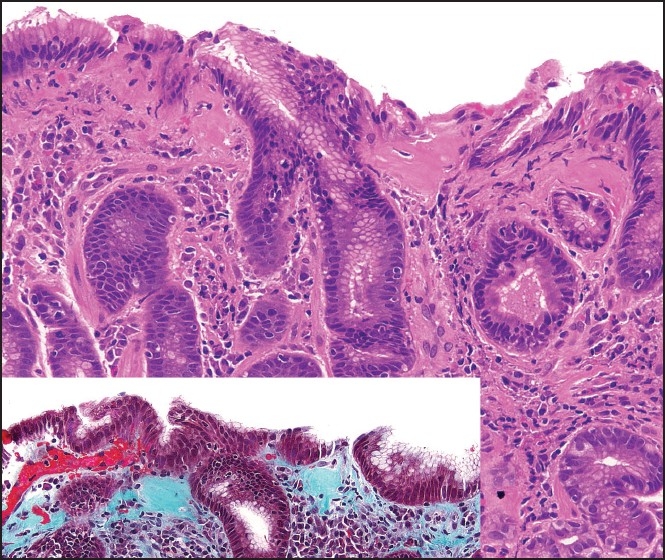
Gastric biopsy with H and E and trichrome stains

## QUESTION

Q1. What is the diagnosis and what is the treatment?

## ANSWER

The gastric biopsies showed lamina propria inflammation with scattered intraepithelial lymphocytes and irregular thickening of the subepithelial collagen band, highlighted by the trichrome stain; these findings are consistent with collagenous gastritis.

The patient was commenced on oral prednisone 20 mg/day for 1 month, and then underwent esophagogastroscopy, which showed noticeable improvement in her gastritis. The steroid dose was tapered over the following 4 weeks to 10 mg/day. Her hemoglobin was measured 2 months after starting therapy and it had increased to 127 g/L with iron supplementation.

Collagenous gastritis is a rare disorder first described in 1989 and characterized by the presence, in the gastric mucosa, of a patchily thickened subepithelial collagen band and intraepithelial inflammatory cells.[1] Patients present with anemia and epigastric pain with or without diarrhea.[1] The endoscopic appearance is variable, and may include nodularity of the gastric corpus mucosa, erythema, erosions, ulcerations, and discrete submucosal hemorrhage.[2]

The cause of collagenous gastritis is unknown. It has been associated with collagenous colitis,[3] lymphocytic colitis,[4] celiac sprue, collagenous sprue, and ulcerative colitis.[5]

Various treatments have been used, including corticosteroids, H2-blockers, proton pump inhibitors, and 5-ASA with variable success. Physicians should consider this rare disorder when more common causes of chronic gastritis, such as *H. pylori* and NSAID gastropathy have been ruled out.
